# Supplementary material for: A Global Screen for Assembly State Changes of the Mitotic Proteome by SEC-SWATH-MS
Source: Cell Syst. 2020 Feb 26;10(2):133–155.e6. doi: 10.1016/j.cels.2020.01.001 (PMC7042714; doi:10.1016/j.cels.2020.01.001)

Q9NYK5 | RM39_HUMAN | MRPL39 C21orf92 MRPL5 RPML5 MSTP003 PRED22
Monomer MW [kDa]: 38.712 Monomer expected elution fraction: 48

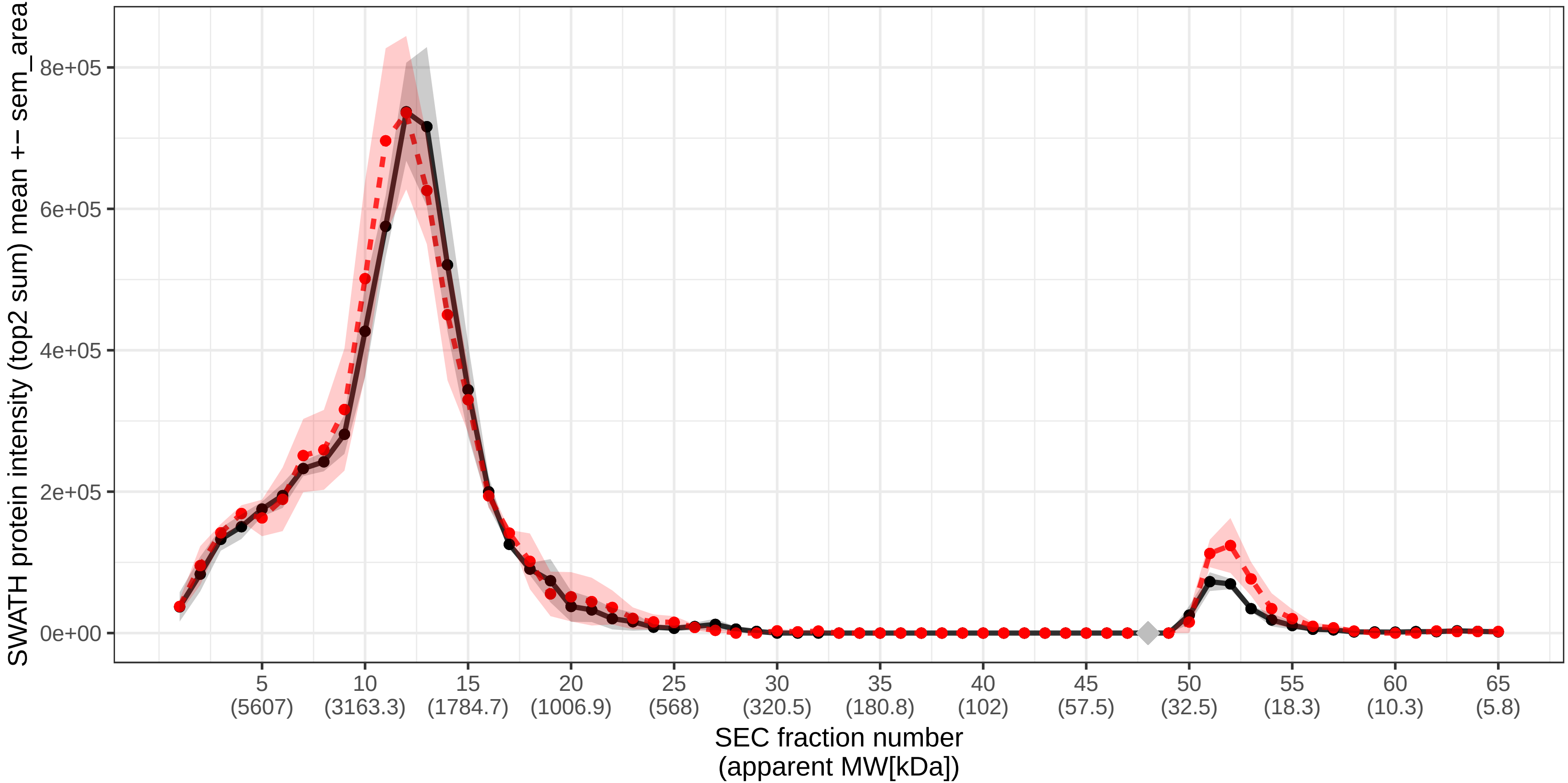

Supplement: Data S1. SEC-SWATH-MS Protein Chromatograms, Related to Figure 1 [file mmc6.zip › SECchrom_Q9NYK5_RM39_HUMAN_MRPL39_C21orf92_MRPL5_RPML5_MSTP.pdf]
